# Epidural bleeding after ACL reconstruction under regional anaesthesia: a case report

**DOI:** 10.1186/1757-1626-2-6732

**Published:** 2009-05-26

**Authors:** Nikolaos T Roidis, Lazaros A Poultsides, Nikolaos E Gougoulias, Paraskevi D Liakou, Theofilos S Karachalios, Konstantinos N Malizos

**Affiliations:** Department of Orthopaedics and Trauma Surgery, University of ThessalyLarissaHellenic Republic

## Abstract

**Introduction:**

Epidural bleeding as a complication of catheterization or epidural catheter removal is often associated with perioperative thromboprophylaxis especially in adult reconstructive surgery.

**Case presentation:**

We report on a case of a 19 years old male athlete that underwent anterior cruciate ligament reconstruction, receiving low molecular weight heparin for thromboprophylaxis and developed an epidural hematoma and subsequent cauda equina syndrome two days after removal of the epidural catheter. An urgent magnetic resonance imaging scan revealed an epidural hematoma from the level of L3 to L4. Emergent decompression and hematoma evacuation resulted in patient's significant neurological improvement immediately postoperatively.

**Conclusion:**

A high index of clinical suspicion and surgical intervention are necessary to prevent such potentially disabling complications especially after procedures on a day-case basis and early patient's discharge.

## Introduction

Spinal epidural haematoma formation as a consequence of epidural catheterization is a rare (e.g. between 1:150000 and 1:190000) but devastating complication [1] and may lead to permanent paraplegia if hematoma evacuation is not performed early after symptom onset [2]. We report on a case of a young male athlete that underwent ACL reconstruction, receiving LMWH for thromboprophylaxis and developed an epidural hematoma and subsequent cauda equina syndrome two days after removal of the epidural catheter.

## Case presentation

**Figure 1.**
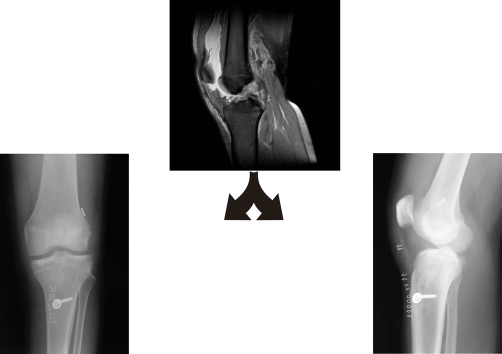
MRI view shows the raptured ACL. Post-op x-rays of the final result.

**Figure 2.**
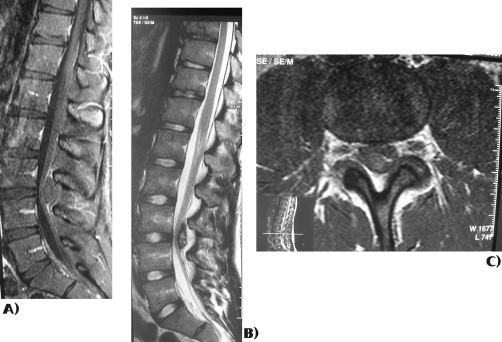
MRI scans show the epidural hematoma at the level of L3-L4.

A 19 years old Caucasian male (soccer player) presented with an anterior cruciate ligament (ACL) rupture of his right knee two months ago. He was admitted in our Department for an arthroscopic ACL reconstruction. His medical history was unremarkable (young athlete) and he reported no medication intake. Preoperative examination did not reveal any disorders and the values of PT (Prothrombin time), aPTT (activated Partial Thromboplastin time), INR and platelet count were normal. An epidural catheter was inserted prior to the operation to provide analgesia postoperatively. Its insertion was quick and easy without any complications as described by the anesthesiologists in charge. The operation was completed uneventfully (75 minutes), regarding both intraoperative anesthesia and the surgery (hamstrings autografts, Endobutton CL, Smith & Nephew, Memphis, Tennessee, USA) and the patient was transferred to the recovery room (Figure 1). During his staying in the recovery room (approximately one hour), he remained hemodynamically stable without any complaints, regaining gradually his muscle strength and sensitivity, and then was transferred to the ward. Anticoagulative treatment started six hour after the end of the operation as in all patients with reconstructive knee surgery and consisted of administration of low molecular weight heparin (LMWH) (Tinzaparine Sodium 4500- anti-Factor Xa IU once daily). Blood loss was not significant and the drain was removed the first postoperative day. Analgesia was provided through the epidural catheter for 48 hours and mobilization started the second postoperative day. The epidural catheter was removed the second postoperative day, 12 hours after the administration of the anticoagulative injection and 12 hours before the next dose, and the patient was placed on oral analgesics when it was needed. The day of surgery and the first postoperative day he reported a minor loss of sensitivity and numbness in both lower extremities which improved till the second postoperative day. At this point, neurological status was normal and mobilization (walking with canes with the knee in an extension brace) continued without any adverse events. The third postoperative day, after the regular morning visit, the patient was discharged being able to ambulate independently without any aid. On the afternoon, he reported moderate loss of sensitivity, perianal and perigenital numbness and muscle weakness in his lower extremities, and low back pain, which improved after the administration of one dose of 500 mg of paracetamol. The fourth postoperative day the patient reported muscle weakness in all muscles of his lower extremities and deteriorating loss of sensitivity (from level L1 to S5) which made his mobilization difficult. He was admitted again in our department; neurologic evaluation revealed at this point muscle weakness in all muscle groups of the lower extremities. According to British Medical Research Council (MRC) scale (Medical Research Council) [3] muscle strength was rated as 3 for iliopsoas, 3 for quadriceps, 3 for hamstrings, 3 for anterior and 3 for the posterior tibial muscles. The patient remained hospitalized, his neurological status worsened rather than improving and the fifth postoperative day, a dose of IV cortisone was administered (30 mg/kg MPS). An epidural hematoma was suspected and the administration of low molecular weight heparin ceased; an urgent MRI scan was ordered which revealed an epidural hematoma from the level of L3 to L4 (Figure 2). Prothrombin time (PT), activated partial thromoblastin time (aPTT) and INR continued to be normal. Immediately, the patient was prepared emergently for the operating room (fifth postoperative day). It is worth mentioning that during patient's preparation in the operating room, just prior to the administration of anaesthesia, a new clinical evaluation showed again worsening of the muscle weakness. In more details, muscle strength was rated as 2 according to MRC scale in all muscle groups: 2 (iliopsoas, quadriceps, hamstrings) or 2 (anterior and posterior tibial muscles). The patient had also lost control of the sphincters, was almost paraplegic, unable even to move legs in bed with significant loss of sensation all over his lower extremities and in perianal and genitary region (cauda equine establishment).

Consequently, an emergent decompression through right fenestration at the level of the hematoma was performed (L3 and L4 vertebrae) without disturbing the facets and the vertebral canal was explored. An extensive clot, which was compressing the dura and its contents had been formed. The hematoma was meticulously evacuated. No bleeding vessels were identified. The wound was closed without fusion of the spine. A bolus dose of methylo-prednizolone (1000 mgr of Solumedrol in one hour) was administered to the patient in the recovery room.

**Figure 3.**
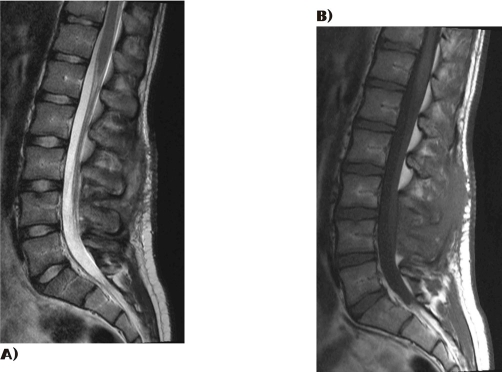
MRI scan show the final result after the decompression of the hematoma at two months.

In the ward, the patient presented significant improvement; he could mobilize his legs without pain and his loss of sensation and numbness were improving continuously. He remained in bed for two days and he was regularly evaluated neurologically. The first day after decompression the patient showed significant neurological improvement with increased muscle strength. Improvement continued and at day 2 post-decompression the patient could move his legs freely in bed and consequently was mobilized. At day 3 postoperatively he could stand alone and make a few steps with a walker without any complaints; his recovery was complete. It is worth mentioning that genitary loss of sensation was the last to disappear. According to MRC scale at day 3 muscle strength was rated as normal for all muscle groups of both lower extremities. The patient was discharged from hospital at day 5 post-fenestration and was re-evaluated 1 week later (day 12 after fenestration). He presented without any neurologic deficit at all and range of motion at the operated right knee ranged from complete extension to 100 degrees of flexion. At 2 months after ACL reconstruction the patient presented with complete muscle strength, and his knee motion ranged from complete extension to 120 degrees of flexion. MRI scan two months post-op demonstrated complete absorption of the haematoma (Figure 3).

## Discussion

Minimal self-limited bleeding in the epidural space may occur in 2.8-11.5% of cases during uncomplicated epidural needle puncture or catheter insertion [4-7]. However, such bleeding may lead to the formation of a compressive epidural haematoma when either a clotting abnormality (spontaneous or drug-induced) exists and/or or difficulty in needle or catheter placement is reported [5]. Risk factors were first described by Vandermeulen et al. [8] including advanced age, female gender, orthopaedic patients, renal insufficiency, ankylosing spondylitis, use of anticoagulants and coagulopathy, difficult catheter insertion and catheter removal. Epidural venous plexi have been suggested as the source of haemorrhage, as epidural veins are more susceptible to rupture after activities that transiently raise venous pressure such as mild exercise, coughing, straining, or other vasalva manoeuvres [9]. Case series from the last few years indicate that spinal epidural haematoma is more common than previously estimated, with a prevalence from 1:100000 in obstetric patients to as high as 1:3600 in female orthopaedic patients [6,10]. Epidural bleeding as a complication of catheterization is often associated with the use of perioperative thromboprophylaxis [5,8,10-16]. Guidelines regarding safe time intervals for epidural catheterization and catheter removal, adopted by many countries, do exist; however they are not evidence based and rely mainly on published reports regarding relevant risks and on drugs pharmacokinetics [12,13]. LMWH administration to prevent deep vein thrombosis has been implicated in the USA for the high-incidence of bleeding after central neural blockade, since the introduction of enoxaparin use in 1993. However similar complication rates in Europe were not observed probably due to the different dosage regimes used in the two continents [17]. As the plasma half-life of LMWH is 4-6 h, 10-12 h is now the recommended safe time interval following LMWH prophylaxis for placement of a central neuraxis or removal of an epidural catheter [5]. Although standard dose LMWHs are associated with a predictable anticoagulant effect considering that the anti-Xa activity after a single bolus of 40 mg enoxaparine for example has nearly returned to baseline after 12 h in patients with normal renal function [18], the risk of unexpected bleeding cannot be excluded [12,19]. It is important to mention that despite existing awareness in a review of 613 cases with spinal haematoma, no clear aetiologic factors were confirmed in about 1/3 of patients [19].

Our patient, a 19 years old young athlete, with a free past medical history, no indication of coagulopathy, normal renal and liver function, no spinal pathology who underwent a typical epidural catheterization and received standard dose LMWH postoperatively was exactly the patient where this rare complication would be more than unexpected. Furthermore, the epidural catheter was removed 12 hours after last dose of LMWH and 12 hours before the next dose was administered. Initially the patient mobilized uneventfully and experienced symptoms of low-back pain, bladder incontinence and sensory and motor dysfunction, worsening in severity two days later. A published case of, similarly to ours, late presentation of epidural bleeding [16], 6 days after catheter removal in an 82-years old patient receiving high dose (0.8 mg/kg twice daily) enoxaparin, was attributed to his altered coagulation status. In two recent reports [11,15] of epidural bleeding after catheterization in three orthopaedic patients, the complication was a consequence of a) preoperative anticoagulant therapy (long-standing dipyridamole [15] and warfarin [11] treatment) in patients undergoing TKA, or b) difficult (multiple punctures) catheterization [15]. In the currently presented case however, no clear risk factors were present, apart from orthopaedic surgical intervention and the use of LMWH. Haematoma formation was attributed to an arterial or venous injury in the vertebral canal, during removal of the catheter, which could not be foreseen. Thromboprophylaxis possibly played a significant role in maintaining bleeding leading gradually to spinal cord compression. To our knowledge, no other case of epidural bleeding in young athletes undergoing an ACL reconstruction has ever been presented.

Surgical intervention and haematoma evacuation has been associated with better outcome [2]. In such a case early diagnosis and treatment is crucial for avoidance of permanent neurologic deficits. High index of suspicion with careful and repeated clinical examination is necessary to evaluate the patient's symptoms, especially if there is gradual worsening, whereas Magnetic Resonance Imaging is the preferred imaging modality that confirms compression in the vertebral canal [20]. In our case, immediate postoperative improvement was evident and complete neurological recovery occurred within a week. The treating surgeon should be however aware of the fact that complete neurological recovery is not always the outcome despite early surgical intervention [2].

## Conclusion

The current report focuses on the relevant rarity of the presented complication, pointing however on the importance of early clinical suspicion and surgical intervention to maximize the possibility of avoiding permanent patient's disability. Although several risk factors have been proposed, the possibility of occurrence in young athletic individuals undergoing sports medicine related reconstructive procedures cannot be excluded. Furthermore, dealing with ACL reconstruction procedures on a day-case basis and early patient's discharge, which is common practice after these procedures, might lead to negligence in diagnosing similar conditions. Close follow-up is thus essential.

## List of abbreviations

aPTT: activated Partial Thromboplastin time
ACL: Anterior cruciate ligament
LMWH: Low molecular weight heparin
MRC: Medical Research Council
MRI: Magnetic resonance imaging
PT: Prothrombine time
TKA: Total knee arthroplasty
